# Elastography evaluation of benign and malignant breast lesions with histopathological data

**DOI:** 10.6026/973206300214880

**Published:** 2025-12-15

**Authors:** Sonali Priyadarsini Reddy, Rajeev Kumar Ranjan, Suresh Kumar Toppo, Anima Ranjni Xalxo, Shahrukh Ansari, Soumik Pal, Mohd Ismail, Manisha Oraon

**Affiliations:** 1 Department of Radiodiagnosis, Rajendra Institute of Medical Science, Ranchi, Jharkhand, India

**Keywords:** Breast lesions, elastography, strain ratio, histopathological correlation, diagnostic accuracy, breast cancer

## Abstract

Breast cancer remains a major health challenge and distinguishing benign from malignant breast lesions is essential to prevent unnecessary procedures and ensure timely treatment. Therefore, it is of interest to evaluate the diagnostic performance of strain elastography in 181 patients with breast lesions. Strain elastography demonstrated significantly higher strain ratios in malignant lesions than benign ones, with a cutoff of ≥3.0 yielding high sensitivity (87.1%) and specificity (90.9%). A strong correlation was observed between elastography findings and histopathology. Strain elastography offers high diagnostic accuracy and can effectively complement routine ultrasound to improve breast lesion characterization.

## Background:

Breast cancer remains the most commonly diagnosed malignancy among women worldwide, with approximately 2.3 million new cases reported annually [1]. In India, breast cancer incidence has been rising steadily, with current estimates suggesting over 150,000 new cases diagnosed each year, accounting for 14% of all cancers in women [2]. The mortality rate remains high, with approximately 90,000 deaths annually, largely attributed to late-stage diagnosis and limited access to advanced healthcare facilities in many regions [3]. Early detection and accurate characterization of breast lesions are critical for improving patient outcomes and reducing mortality [4]. Conventional imaging modalities, including mammography and ultrasound (US), form the cornerstone of breast lesion evaluation. However, these techniques have inherent limitations. Mammography has reduced sensitivity in dense breast tissue, which is particularly common in younger Asian women [5]. While conventional US demonstrates high sensitivity (90-95%) for detecting breast lesions, its specificity remains relatively low (35-75%), leading to unnecessary biopsies for benign lesions [6]. Elastography has emerged as a promising adjunctive imaging technique that evaluates tissue elasticity, providing additional information beyond conventional morphological assessment [7]. The technique is based on the principle that malignant tissues are typically stiffer than benign tissues due to increased cellularity, desmoplastic reaction and architectural distortion [8]. Two main elastography techniques are currently available: strain elastography and shear-wave elastography. Strain elastography assesses tissue deformation under external compression, while shear-wave elastography measures the propagation speed of shear waves generated by acoustic radiation force [9, 10]. Similarly, a systematic review by Zou *et al.* found that adding elastography to conventional US improved specificity from 61% to 79% while maintaining sensitivity at 89% [11]. However, there remains considerable variability in reported diagnostic performance across different studies, with sensitivity ranging from 65% to 98% and specificity from 46% to 98% [12]. Despite these promising results, several research gaps persist. Studies have been conducted in Western populations, with limited data from Asian countries where breast cancer presents at a younger age and with different pathological characteristics [13]. The optimal strain ratio cutoff value for differentiating benign and malignant lesions remains controversial, with reported values ranging from 2.27 to 4.5 across different studies [14]. Therefore, it is of interest to evaluate the diagnostic performance of strain elastography in differentiating benign and malignant breast lesions in an Indian population and to validate its effectiveness through correlation with histopathological findings.

## Materials and Methods:

## Study design and setting:

This hospital-based cross-sectional study was conducted in the Department of Radiodiagnosis at Rajendra Institute of Medical Sciences (RIMS), Ranchi, India, over a 12-month period from April 2024 to January 2025. The study protocol was approved by the Institutional Ethics Committee (Certificate No. 126 IEC RIMS dated 06/04/2024) and written informed consent was obtained from all participants.

## Sample size:

The sample size was calculated based on the prevalence data from GLOBOCAN 2020, which reported a breast cancer prevalence of 181 cases in the study region. Therefore, 181 patients were selected for the study to ensure adequate representation of both benign and malignant lesions.

## Study population:

The study included patients admitted to the Surgery IPD at RIMS, Ranchi, with breast lesions detected through clinical examination, ultrasound, or other imaging modalities. These patients were subsequently referred to the Department of Radiodiagnosis for strain elastography evaluation.

## Inclusion criteria:

[1] Adult female patients (over 18 years of age)

[2] Patients with breast lesions detected through clinical examination, ultrasound, or other imaging techniques

[3] Patients who provided written informed consent

## Exclusion criteria:

[1] Patients with previous breast surgeries that may affect elastographic results

[2] Patients who had undergone prior interventions (e.g., breast biopsy or localization procedures) that might impact elastography findings

[3] Patients with poor-quality elastographic images due to technical limitations, artifacts, or insufficient image acquisition

[4] Patients with missing or insufficient data required for analysis, including incomplete elastography or histopathological information

[5] Pregnant or lactating patients due to hormonal changes that can influence breast tissue characteristics and elastography results

[6] Patients unable to provide informed consent

## Imaging protocol:

All patients underwent standard ultrasound (B-mode) and strain elastography using a PHILIPS AFFINITI 70 G machine with a high-frequency (8-12 MHz) linear probe. Following a physical breast examination, any identified breast issues were documented. During imaging, patients lay in the supine position with the opposite side slightly rotated and the arm on the same side raised above their head. Elastography images were acquired in this same position. Standard ultrasound included scanning the whole breast and axillary tail region from the nipple outward. For any breast lesions found, images were obtained in both longitudinal and transverse planes. These lesions were evaluated based on their location, size, shape, edge characteristics (borders and margins), orientation (whether they were taller or wider than they were deep), echogenicity compared to surrounding tissue, presence of posterior acoustic shadowing, presence of calcifications and effects on surrounding tissue.

## Lesions were categorized using the BI-RADS classification for conventional breast ultrasound as follows:

[1] Category 1: Negative

[2] Category 2: Benign lesions

[3] Category 3: Probably benign

[4] Category 4 (a, b, c): Suspicious

[5] Category 5: Highly suggestive of malignancy

[6] Category 6: Pathologically confirmed malignant lesions

## Strain Elastography Technique:

The elasticity score, based on the Tsukuba scoring system proposed by Itoh *et al.* was used to classify lesions:

[1] Score 1: Strain throughout the entire hypoechoic lesion (green color)

[2] Score 2: Partial strain seen in the lesion (mixture of green and blue)

[3] Score 3: Strain only seen peripherally (green around the edges)

[4] Score 4: No strain within the lesion (blue)

[5] Score 5: No strain in the lesion or surrounding area (both lesion and surrounding area in blue)

Higher elasticity scores indicated stiffer tissue. Additionally, strain ratio (SR) was calculated by comparing the strain within the lesion to that in the surrounding fatty tissue. A region of interest (ROI) was placed within the lesion and another in the adjacent fatty tissue at the same depth. The SR was calculated as the average strain in the fatty tissue divided by the average strain in the lesion.

## Histopathological examination:

Two experienced pathologists examined all breast lesions histopathologically. Tissue samples were obtained using various methods, including fine needle aspiration cytology (FNAC), core biopsy, surgical excision, or radical surgery. The histopathological results were considered the gold standard for diagnosis and were correlated with the ultrasound elastography findings, specifically the strain ratio and elasticity score.

## Statistical analysis:

Numerical data were presented as mean ± standard deviation (SD). To determine significant differences between groups, Student's t-test was used after confirming normal distribution. Categorical data were expressed as counts and percentages. Relationships between variables were analyzed using Fisher's exact test, Student's t-test and the Chi-square test. A p-value < 0.05 was considered statistically significant. Statistical analyses were performed using SPSS version 20.0 (IBM Corp., Armonk, NY, USA) and MS Excel 2010.

## Results:

A total of 181 female patients with breast lesions were included in the study. The age distribution of the participants is presented in Table 1 (see PDF). The predominant age group was 51-60 years (26.5%), followed by 31-40 years (22.0%) and 41-50 years (20.1%). The youngest participant was 18 years old and the oldest was 70 years old, with a mean age of 41.3 ± 12.7 years. Left breast lesions accounted for 51.4% (n=93) of cases, while right breast lesions constituted 48.6% (n=88). The majority of patients (73.4%, n=133) presented with solitary lesions, whereas multiple lesions were observed in 26.5% (n=48) of the cohort. Regarding lesion characteristics, solid lesions were the predominant type (86.1%, n=156), followed by mixed lesions (10.0%, n=18) and cystic lesions (3.9%, n=7). According to the BI-RADS classification, the most prevalent category was category 2 (42%, n=76), followed by category 4 (22.6%, n=41), category 5 (21.4%, n=39) and category 3 (14%, n=25). No lesions were categorized as either BI-RADS 1 or BI-RADS 6. Histopathological evaluation revealed benign pathology in 61.4% of patients (n=111) and malignant lesions in 38.6% (n=70). Among benign lesions, fibroadenoma was the most frequent finding (61.3%, n=68), followed by breast abscess (16.2%, n=18), benign cyst (6.3%, n=7), granulomatous mastitis (5.4%, n=6), lipoma (8.1%, n=9), benign papillary lesion (0.9%, n=1), tuberculous lymphadenitis (0.9%, n=1) and usual ductal hyperplasia (0.9%, n=1). Among malignant lesions, invasive ductal carcinoma predominated (57.1%, n=40), followed by ductal carcinoma in situ (33%, n=23), phyllodes tumor (7.1%, n=5), lobular carcinoma (1.4%, n=1) and atypical ductal hyperplasia (1.4%, n=1) (Table 2 - see PDF). The mean strain ratio for benign and malignant lesions was 2.45±1.21 and 3.63±1.57, respectively. Student's t-test revealed a statistically significant difference in strain ratio values between benign and malignant lesions (p < 0.05). Following strain ratio calculation for each lesion, correlation with histopathological findings was performed. A strain ratio ≥ 3.0 was designated indicative of malignancy, while a ratio < 3.0 was considered indicative of benignity. Strain elastography demonstrated the ability to accurately identify 61 out of 70 malignant lesions (87.1%). Furthermore, 101 out of 111 benign lesions (90.1%) were correctly classified based on strain ratio evaluation. Chi-square analysis revealed a statistically significant correlation between strain ratio and histopathological outcome (p<0.05). The diagnostic performance of strain ratio, in reference to histopathological diagnosis, is presented in Table 3 (see PDF). The calculated specificity, sensitivity, positive predictive value (PPV), negative predictive value (NPV) and overall accuracy were 90.9%, 87.1%, 89.5%, 87.1% and 89.0%, respectively.

## Discussion:

This study evaluated the diagnostic performance of strain elastography in differentiating benign and malignant breast lesions in an Indian population, with histopathological examination serving as the gold standard. Our findings demonstrate that strain elastography is a valuable adjunct to conventional ultrasound, with high sensitivity (87.1%) and specificity (90.9%) in distinguishing malignant from benign lesions. The mean strain ratio was significantly higher in malignant lesions (3.63±1.57) compared to benign lesions (2.45±1.21), which aligns with the established principle that malignant tissues are typically stiffer than benign tissues due to increased cellularity, desmoplastic reaction and architectural distortion [15, 16, 17, 18-19]. Our slightly higher specificity may be attributed to the standardized protocol and rigorous quality control measures implemented in our study. The optimal strain ratio cutoff value for differentiating benign and malignant lesions remains controversial, with reported values ranging from 2.27 to 4.5 across different studies [20]. In our study, a cutoff value of 3.0 provided the best balance between sensitivity and specificity. This is slightly lower than the cutoff of 3.67 reported by Gheonea *et al.* [17] but higher than the 2.77 cutoff suggested by Zhi *et al.* [21]. These variations may be due to differences in patient populations, equipment, measurement techniques and the prevalence of specific lesion types in different studies. The high negative predictive value (87.1%) observed in our study suggests that strain elastography could be particularly useful in reducing unnecessary biopsies for lesions that are likely benign. This is clinically significant, as unnecessary biopsies not only increase healthcare costs but also cause patient anxiety and potential complications [22]. By integrating strain elastography into routine breast imaging protocols, clinicians could potentially reduce the number of benign lesions sent for biopsy while maintaining high sensitivity for malignant lesions. Our findings also demonstrated a statistically significant correlation between strain ratio and histopathological outcome (p<0.05), further validating the utility of elastography as a reliable diagnostic tool. This correlation is consistent with previous studies that have shown good agreement between elastographic findings and histopathological results [23, 24]. The distribution of lesion types in our study reflects the typical pattern seen in Indian populations, with fibroadenoma being the most common benign lesion (61.3%) and invasive ductal carcinoma the most common malignancy (57.1%) [25]. The relatively high proportion of malignant lesions (38.6%) in our study may be attributed to the tertiary care setting of our institution, which tends to receive more complex cases [26]. Despite the promising results, our study has several limitations. First, the cross-sectional design does not allow for long-term follow-up of patients. Second, our study was conducted at a single tertiary care center, which may limit the generalizability of our findings to different healthcare settings. Third, we did not evaluate inter-observer variability in elastography interpretation, which could affect the reproducibility of our results. Fourth, the relatively small sample size may have limited our ability to detect subtle differences in diagnostic performance across different lesion types. Future studies should address these limitations by conducting larger multicenter trials with long-term follow-up, evaluating inter-observer variability and comparing different elastography techniques (strain vs. shear-wave) in the same patient population. Additionally, research should focus on developing standardized protocols for elastography performance and interpretation to ensure consistent results across different institutions and operators. In conclusion, our study demonstrates that strain elastography is a valuable adjunct to conventional ultrasound for differentiating benign and malignant breast lesions, with high diagnostic accuracy. Its integration into routine breast imaging protocols could reduce unnecessary biopsies while maintaining high sensitivity for malignant lesions, ultimately improving patient care and optimizing healthcare resource utilization.

## Conclusion:

Strain elastography shows strong diagnostic ability in distinguishing benign from malignant breast lesions, with excellent sensitivity, specificity and overall accuracy. Its significant correlation with histopathological findings supports its role as a reliable, non-invasive adjunct to conventional ultrasound. Incorporating elastography into routine breast imaging can reduce unnecessary biopsies, optimize resource use and improve diagnostic confidence, particularly in resource-limited settings.
